# Lipopolysaccharides Enhance Epithelial Hyperplasia and Tubular Adenoma in Intestine-Specific Expression of *kras^V^*^12^ in Transgenic Zebrafish

**DOI:** 10.3390/biomedicines9080974

**Published:** 2021-08-07

**Authors:** Jeng-Wei Lu, Yuxi Sun, Pei-Shi Angelina Fong, Liang-In Lin, Dong Liu, Zhiyuan Gong

**Affiliations:** 1 Department of Biological Sciences, National University of Singapore, Singapore 117543, Singapore; e0437708@u.nus.edu (Y.S.); a0131047_angelina@u.nus.edu (P.-S.A.F.); 2 Department of Clinical Laboratory Sciences and Medical Biotechnology, National Taiwan University, Taipei 10048, Taiwan; lilin@ntu.edu.tw; 3 Department of Biology, Southern University of Science and Technology, Shenzhen 518055, China; liud@sustech.edu.cn; 4 Department of Laboratory Medicine, National Taiwan University Hospital, Taipei 10048, Taiwan

**Keywords:** colorectal cancer, dextran sulfate sodium, lipopolysaccharides, *kras^V^*^12^, intestinal tumor, transgenic zebrafish

## Abstract

Intestinal carcinogenesis is a multistep process that begins with epithelial hyperplasia, followed by a transition to an adenoma and then to a carcinoma. Many etiological factors, including *KRAS* mutations and inflammation, have been implicated in oncogenesis. However, the potential synergistic effects between *KRAS* mutations and inflammation as well as the potential mechanisms by which they promote intestinal carcinogenesis remain unclear. Thus, the objective of this study was to investigate the synergistic effects of *kras^V^*^12^, lipopolysaccharides (LPS), and/or dextran sulfate sodium (DSS) on inflammation, tumor progression, and intestinal disorders using transgenic adults and larvae of zebrafish. Histopathology and pathological staining were used to examine the intestines of *kras^V^*^12^ transgenic zebrafish treated with LPS and/or DSS. LPS and/or DSS treatment enhanced intestinal inflammation in *kras^V^*^12^ transgenic larvae with concomitant increases in the number of neutrophils and macrophages in the intestines. The expression of *kras^V^*^12^, combined with LPS treatment, also enhanced epithelial hyperplasia and tubular adenoma, demonstrated by histopathological examinations and by increases in cell apoptosis, cell proliferation, and downstream signaling of phosphorylated AKT serine/threonine kinase 1 (AKT), extracellular-signal-regulated kinase (ERK), and histone. We also found that *kras^V^*^12^ expression, combined with LPS treatment, significantly enhanced changes in intestinal morphology, specifically (1) decreases in goblet cell number, goblet cell size, villi height, and intervilli space, as well as (2) increases in villi width and smooth muscle thickness. Moreover, *kras^V^*^12^ transgenic larvae cotreated with DSS and LPS exhibited exacerbated intestinal inflammation. Cotreatment with DSS and LPS in *kras^V^*^12^-expressing transgenic adult zebrafish also enhanced epithelial hyperplasia and tubular adenoma, compared with wild-type fish that received the same cotreatment. In conclusion, our data suggest that *kras^V^*^12^ expression, combined with LPS and/or DSS treatment, can enhance intestinal tumor progression by activating the phosphatidylinositol-3-kinase (PI3K)/AKT signaling pathway and may provide a valuable in vivo platform to investigate tumor initiation and antitumor drugs for gastrointestinal cancers.

## 1. Introduction

Colorectal cancer (CRC) ranks third in terms of global cancer incidence. This disease causes more than 600,000 deaths every year, and the number of affected individuals continues to increase around the world [1,2,3]. In recent years, the incidence and mortality of CRC have also continued to rise rapidly. Between 2007 and 2016, the high mortality rates for CRC were 35% (United States), 45% (Europe), and 47.8% (worldwide) [4,5,6]. Furthermore, the high recurrence and low survival rates of this disease seriously affect patient quality of life [7].

RAS proteins, which are GTPases that regulate the RAS signaling pathway and control cell proliferation and cell survival, are often mutated in human cancers [8]. CRC results from a multistep process of carcinogenesis that is caused by the accumulation of genetic mutations as well as changes in signal transduction pathways. Approximately 35% of CRC cases are caused by genetic mutations, with KRAS and NRAS gene mutations respectively accounting for 40% and 5% of these cases caused by genetic mutations [9,10]. Previous research reported that 85% of KRAS gene mutations occur in codons 12 and 13 of exon 2 [11]. During carcinogenesis, the activation of KRAS proteins triggers tumor initiation and accelerates tumor growth. KRAS mutations have been detected in both early- and late-stage CRC patients, which indicates that KRAS mutations likely occur in the early stages of tumor development [12]. 

CRC development is also related to the composition of the gut microbiota because it involves immune, structural, and metabolic processes [13,14]. Importantly, the destruction of gut microbiota and/or imbalance in gut microbiota composition may be related to CRC formation [15,16]. Toll-like receptor 4 (TLR4) can bind to lipopolysaccharides (LPS), which activate the nuclear transcription factor kappa B (NF-κB) signaling pathway, from Gram-negative bacteria [17]. This in turn induces other innate immune responses as well as proinflammatory gene expression and recruitment of the adaptive immune system [18]. Notably, LPS strongly stimulates innate immune signaling, thereby impairing intestinal homeostasis and normal host physiology. LPS thus plays a vital role in promoting the progression and metastasis of CRC [17,19,20]. In addition, exposure to the inflammatory agent dextran sulfate sodium (DSS) for 1 week in combination with a single treatment of azoxymethane can accelerate the induction of CRC in rodents [21]. 

Zebrafish are considered an excellent animal model for studying human gastrointestinal cancer [22,23]. In terms of histological morphology and the expression profiles of dysregulated genes, many zebrafish models of intestinal diseases and tumors are similar to human disease states. As a vertebrate, the zebrafish has a highly conserved anatomical structure and homologous organs with higher vertebrates, including humans, and most of the signal pathways that control apoptosis, proliferation, differentiation, and movement are also highly conserved between zebrafish and human. Furthermore, the high degree of homology and oncogenes and tumor suppressor genes also reveal that the carcinogenic mechanism between zebrafish and higher vertebrates is also highly conserved. Zebrafish studies also show the activation of carcinogenic signaling pathways, such as RAS, tumor protein p53 (Tp53), and Wnt/β-catenin pathways, which plays an important role in CRC. It has been found that the tumor biology, intestinal disorders caused by carcinogens, and the morphological pattern of tumors are highly similar between zebrafish and humans [23]. Intestinal tumors can be induced by 7,12-dimethylbenz(a)anthracene (DMBA). Moreover, in adenomatous polyposis coli (*apc*) mutated zebrafish [24], intestinal diseases and tumors can be driven by inducible *kras^V^*^12^ or by the continuous expression of *kras^G^*^12^ or *Helicobacter. pylori* virulence factor cagA (*cagA*) with wild-type (WT) or *tp53* mutated zebrafish [25,26,27,28]. The zebrafish xenograft model also provides an excellent platform for studying CRC tumor metastasis and drug screening [29,30,31]. Previous research has shown zebrafish patient-derived xenografts (zPDX) derived from surgically resected human CRC samples and treated with the same treatment administered to the donor patient. This study provides a proof-of-concept experiment that can compare the CRC patient and zPDX with chemotherapy and biotherapy response [30].

In this study, we report the potential synergy between intestine-specific overexpression of *kras^V^*^12^ [27] and LPS and/or DSS treatment in the development and progression of intestinal tumors in transgenic zebrafish. We used histopathology and pathological staining to examine the potential effects of *kras^V^*^12^ and LPS and/or DSS treatment on intestinal disease and intestinal tumors. In so doing, a further goal was to investigate the potential mechanisms that underlie these disorders.

## 2. Materials and methods

### 2.1. Zebrafish Husbandry

Zebrafish embryos and larvae as well as adult zebrafish (*Danio rerio*) were maintained according to established protocols described in our previous studies [27,32,33,34]. Tg(ifabp:EGFP-kras^V12^), Tg(lyz:DsRed), and Tg(mpeg1:mCherry) transgenic zebrafish embryos and larvae as well as adult zebrafish were maintained at 28 °C under continuous water flow and a 14 h light and 10 h dark cycle. The intestine-specific *kras^V^*^12^ transgenic zebrafish were generated as previously described [27], and wild-type (WT) zebrafish were used as a control. All experiments involving zebrafish were approved by the Institutional Animal Care and Use Committee (IACUC) of the National University of Singapore and National Taiwan University.

### 2.2. Mifepristone and Chemical Treatments of Zebrafish

Each larva treatment group included 20 larvae, which were maintained in six-well plates. Each well contained 1X E3 medium and mifepristone (catalog number: M8046; Sigma-Aldrich, St. Louis, MO, USA), DSS (catalog number: D8906; Sigma-Aldrich, St. Louis, MO, USA), and/or LPS (catalog number: L4391; Sigma-Aldrich, St. Louis, MO, USA). For the DSS and/or LPS treatment groups, larvae were treated with 0.05% DSS and/or 40 ng/mL of LPS for 2 or 3 days postinduction (dpi). To induce *kras^V^*^12^ expression, larvae were also treated with 4 μM mifepristone for 2 or 3 dpi. Larvae were incubated at 28 °C, and mortality was determined daily. The 1X E3 medium, fresh mifepristone, and chemicals were treated every other day.

Each adult zebrafish treatment group was maintained in a 5 L tank at room temperature, and all zebrafish were fed normally. At 4 weeks postinduction (wpi), samples were collected to investigate long-term treatment effects. For the DSS and/or LPS treatment groups, 4-month-old zebrafish were treated with 0.00625% DSS and/or 40 ng/mL of LPS for 4 wpi. To induce *kras^V^*^12^ expression, zebrafish were exposed to 2 μM mifepristone at 4 wpi. The mortality of adult zebrafish was determined daily, and water, fresh mifepristone, and chemicals were treated every other day.

### 2.3. Tissue Collection and Histopathology of Zebrafish Intestines

Control and transgenic zebrafish were euthanized at 5 months of age using 0.02% tricaine (catalog number: E10521; Sigma-Aldrich, St. Louis, MO, USA). Zebrafish intestines were then collected and fixed in 10% neutral buffered formalin solution (catalog number: HT501128; Sigma-Aldrich, St. Louis, MO, USA) overnight, embedded in paraffin, sectioned into 4 μm sections, and then mounted on poly-L-lysine-coated slides at different time points following mifepristone induction and chemical treatment. The slides were stored in slide boxes at room temperature.

Cytological analysis was also performed on the collected zebrafish intestines. After hematoxylin and eosin (H&E) staining was completed, intestinal histopathology was assessed via a single-blind evaluation of all samples. All intestine tissue evaluations were based on four consecutive sagittal serial sections, which composed the entire intestinal tract, anterior to posterior. Specifically, tissue samples were evaluated for epithelial hyperplasia, dysplasia, and tumors according to previously described diagnostic criteria [26,27].

### 2.4. Immunofluorescence (IF) and Periodic Acid–Schiff (PAS) Staining

The 4 μm zebrafish sections were dewaxed using histoclear (catalog number: H2779; Sigma-Aldrich, St. Louis, MO, USA) and hydrated in an ethanol gradient and Milli-Q water for 10 min, respectively. For antigen retrieval, endogenous peroxidase activity was blocked by heating the slides at 100 °C for 20 min in 10 mM citric acid buffer (catalog number: C9999; Sigma-Aldrich, St. Louis, MO, USA). This was followed by blocking with 3% H_2_O_2_ for 15 min. The slides were then washed three times with 1X phosphate-buffered saline (catalog number: P3813; Sigma-Aldrich, St. Louis, MO, USA) with 0.1% Tween 20 (catalog number: 9005-64-5; Sigma-Aldrich, St. Louis, MO, USA) (PBST) for 5 min. Following this, slides were blocked again using 5% bovine serum albumin (BSA) (catalog number: A2153; Sigma-Aldrich, St. Louis, MO, USA) at room temperature for 30 min and then incubated with specific primary antibodies in a humidifying chamber at 4 °C overnight. After being washed with 1X PBST, the slides were incubated with conjugated fluorescent secondary antibodies and then incubated with 4′,6-diamidino-2-phenylindole (DAPI) (catalog number: D9542 Sigma-Aldrich, St. Louis, MO, USA) for 10 min. Finally, the slides were dehydrated, cleared, and mounted. To determine the specificity of primary antibodies for IF staining, we performed experiments using both appropriate positive (from a previously known positive case) and negative controls (slides not incubated with primary antibodies).

For PAS staining, tissues were also dewaxed using histoclear and hydrated in an ethanol gradient and Milli-Q water for 10 min, respectively. Following this, staining was performed using the Periodic Acid–Schiff Stain Kit (catalog number: 24200-1; Polysciences, Inc., Warrington, PA, USA) to detect goblet cells. Goblet cells were evaluated according to the number of villi. Finally, the slides were dehydrated, cleared, and mounted and then examined using light or fluorescent microscopy.

### 2.5. Statistical Analysis

Differences between experimental and control groups were analyzed by two-tailed Student’s t-test and one-way ANOVA. Plot survival curves were derived using the Kaplan–Meier method, and log-rank tests were used to examine differences between experimental and control groups. *p*-Values of less than 0.05 were considered statistically significant.

## 3. Results

### 3.1. Effects of LPS or DSS Treatment on Intestinal Inflammation in kras^V12^ Transgenic Zebrafish Larvae

**Figure 1:**
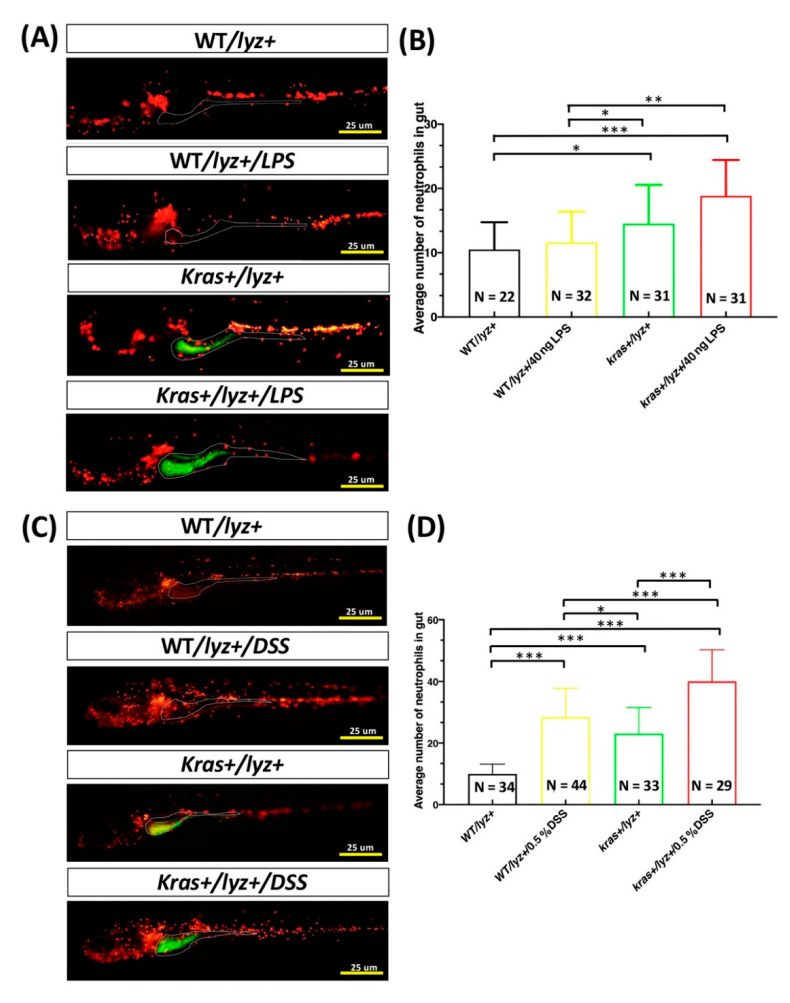

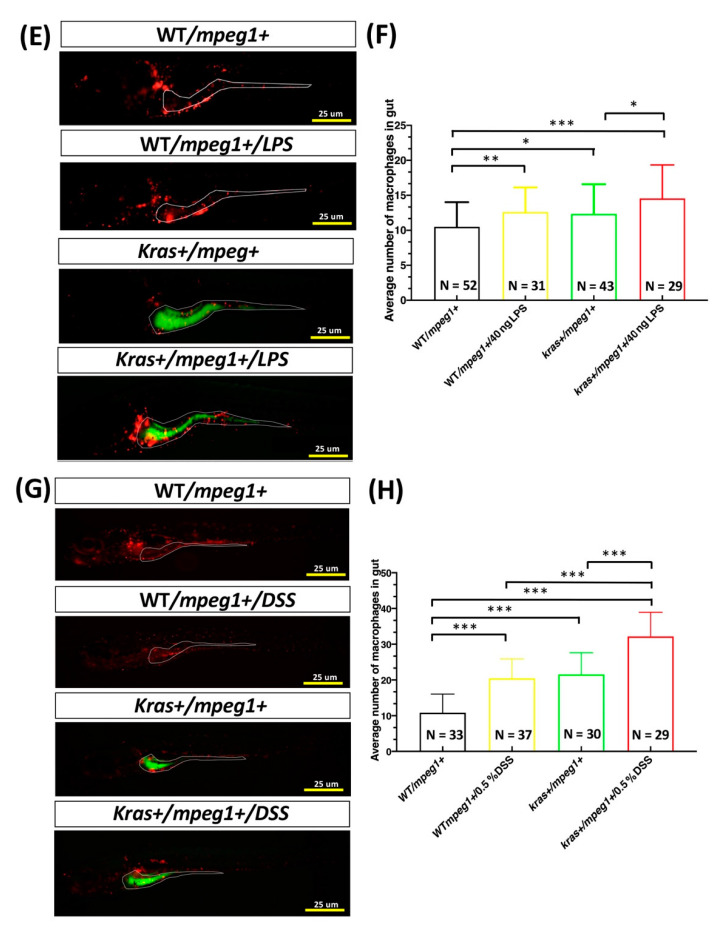
DSS or LPS enhances the increase in intestinal neutrophils and macrophages in kras+/lyz+ and kras+/mepg1+ zebrafish larvae. (**A**,**C**,**E**,**G**) Fluorescence of neutrophils and macrophages in the intestine. (**B**,**D**,**F**,**H**) Quantification of the number of positive cells as revealed by fluorescence of neutrophils or macrophages. Differences among the variables were assessed using Student’s t-tests. Statistical significance: * *p* < 0.05, ** *p* < 0.01, *** *p* < 0.001. Scale bar: 25 μm.

To examine the effects of intestinal inflammation, the following groups of zebrafish larvae from 4 to 6 or 7 dpf were treated with 40 ng/mL of LPS or 0.5% DSS in addition to treatment with 4 μM of mifepristone (to induce *kras^V^*^12^ expression): WT/lyz+/LPS, kras+/lyz+/LPS, WT/mpeg1+/LPS, kras+/mpeg1+/LPS, WT/lyz+/DSS, kras+/lyz+/DSS, WT/mpeg1+/DSS, kras+/mpeg1+/DSS. WT/lyz+, kras+/lyz+, WT/mpeg1+, and kras+/mpeg1+ zebrafish larvae without treatments were used as controls. All larvae from each group were imaged. Neutrophils and macrophages in intestines were respectively counted based on the fluorescence of DsRed or mCherry. We observed significant increases in neutrophils in the kras+/lyz+/LPS and kras+/lyz+/DSS larva groups compared with WT/lyz+, WT/lyz+ with LPS or DSS treatment, and kras+/lyz+ (Figure 1A–D) larva groups. We also observed significant increases in macrophages in the kras+/mpeg1+/LPS and kras+/mpeg1+/DSS larva groups compared with the kras+/mpeg1, WT/mpeg1+ with DSS treatment, and kras+/mpeg1+ (Figure 1E–H) larva groups.

### 3.2. Phenotype of Intestinal Tumors Induced by Sustained Expression of kras^V12^ with LPS Treatment in Transgenic Zebrafish

**Figure 2:**
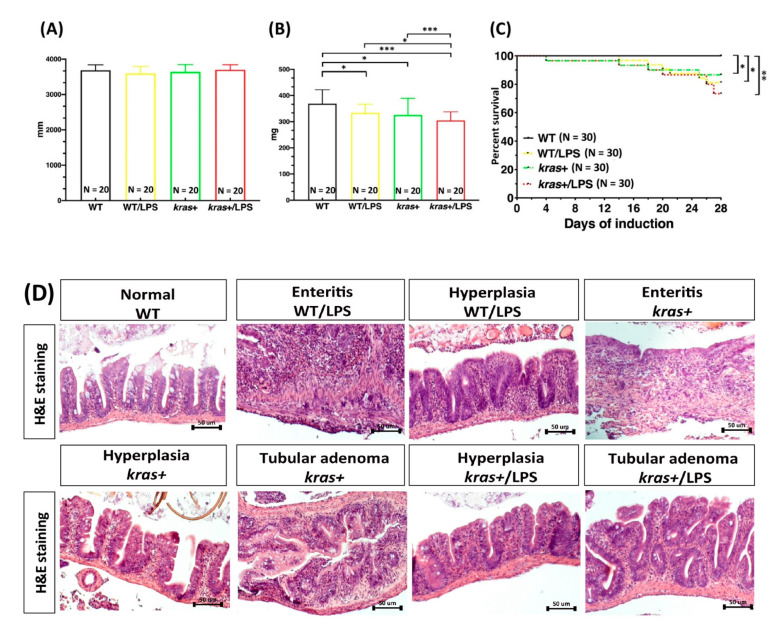

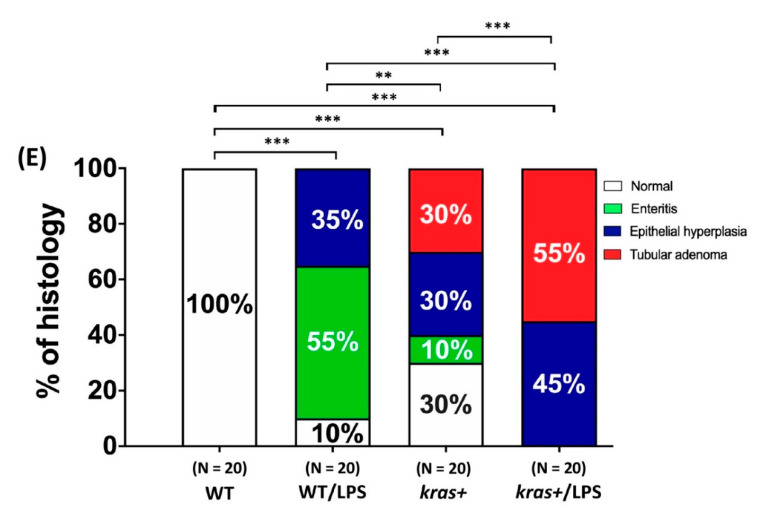
Synergistic effect of *kras^V^*^12^ expression and LPS treatment in intestinal tumorigenesis. Four-month-postfertilization WT and kras+ zebrafish were cotreated with 2 μM of mifepristone and 40 ng/mL of LPS for 4 weeks, and samples were then collected for gross observations and histological analyses. There were four experimental groups: WT, WT/LPS, kras+, and kras+/LPS. (**A**,**B**) Body length and body weight. (**C**) Survival curves. (**D**) Examples of normal intestines, enteritis, hyperplasia, and tubular adenoma as revealed by H&E-stained sections of the intestine. (**E**) Summary of intestinal histological abnormalities observed in the four experimental groups. These data were generated from results of a blinded histological analysis (WT, N = 20; WT/LPS, N = 20; kras+, N = 20; kras+/LPS, N = 20). Differences among the variables were assessed using Student’s t-tests or one-way ANOVA. Statistical significance: * *p* < 0.05, ** *p* < 0.01, *** *p* < 0.001. Scale bar: 50 μm.

Results of previous studies demonstrated that treating adult-stage zebrafish with the colitogenic agent DSS can enhance intestinal tumorigenesis in *kras^V^*^12^-expressing transgenic zebrafish [27]. To examine whether LPS could also enhance tumorigenesis in *kras^V^*^12^ adult transgenic zebrafish through the induction of inflammation, heterozygous *kras^V^*^12^ transgenic zebrafish were cotreated with 2 μM of mifepristone and 40 ng/mL of LPS for 4 weeks at 4 months postfertilization (mpf). We found no significant difference in body length between *kras^V^*^12^ transgenic zebrafish treated with LPS (kras+/LPS) and the control group (Figure 2A). However, LPS treatment did lead to significantly reduced body weights in WT/LPS and kras+/LPS adult zebrafish compared with WT, WT/LPS, and kras+ zebrafish (Figure 2B). Furthermore, by 4 wpi, four of the kras+ zebrafish and eight of the kras+/LPS zebrafish had died. During the same period, five WT/LPS and no WT control zebrafish died (Figure 2C).

At 4 wpi, we also evaluated the entire intestinal tract of all adult zebrafish for enteritis, epithelial hyperplasia, and the presence of tumors (Figure 2D). Intestinal samples were collected from the WT, WT/LPS, kras+, and kras+/LPS groups, and histological examinations were then performed. While all WT zebrafish showed normal intestinal histology, LPS treatment respectively caused enteritis and hyperplasia in 55% and 35% of WT zebrafish. Conversely, kras+ zebrafish treated with mifepristone exhibited inflammation (10%), hyperplasia (30%), and tubular adenoma (30%). LPS treatment in kras+ zebrafish further increased the prevalence of abnormalities: 45% and 55% of adult kras+/LPS zebrafish respectively showed hyperplasia and tubular adenoma. These observations suggest that *kras^V^*^12^ expression combined with LPS treatment significantly enhanced intestinal tumors in adult zebrafish compared with untreated WT zebrafish, WT zebrafish treated with LPS, and kras+ zebrafish that did not undergo LPS treatment (Figure 2E). 

### 3.3. Induction of kras^V12^ Expression with LPS Treatment Decreased the Number of Goblet Cells, Goblet Cell Size, Villi Height, and Intervilli Space and Increased Villi Width and Smooth Muscle Thickness in Fish Intestines

**Figure 3:**
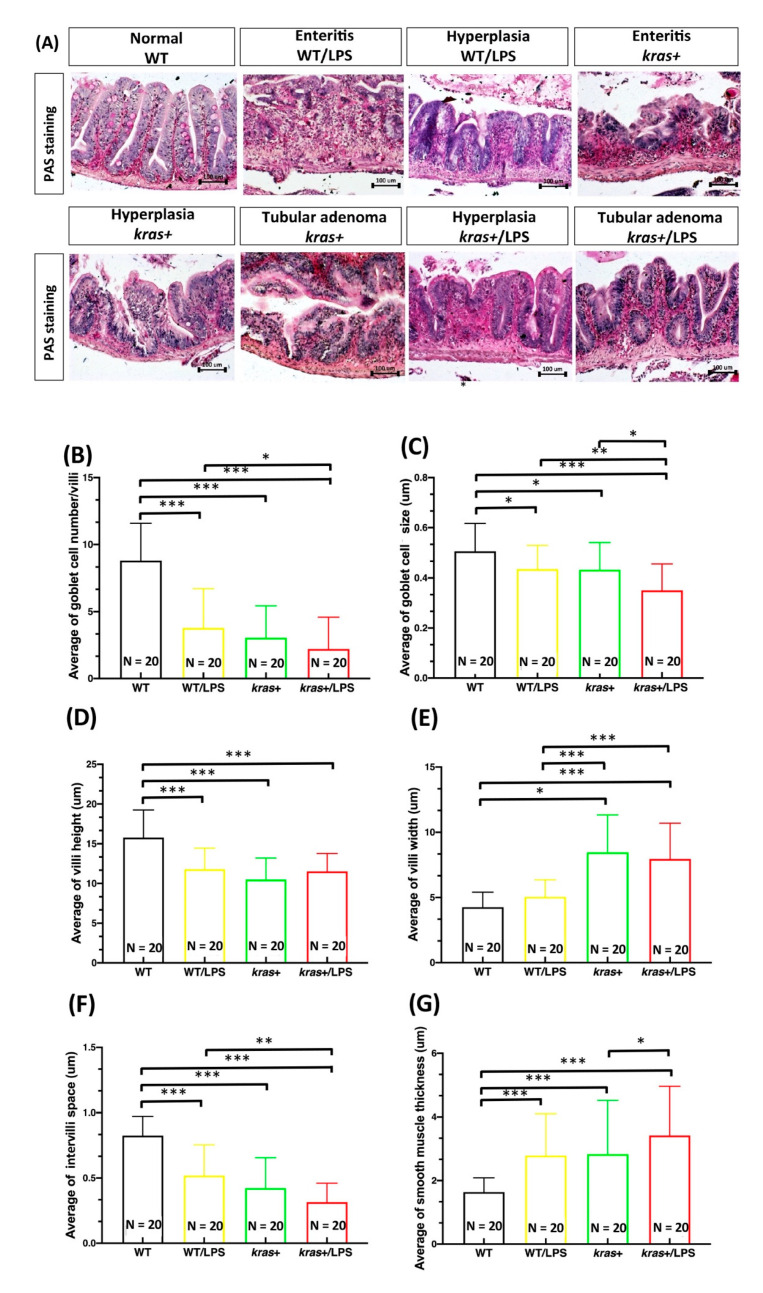
Expression of *kras^V^*^12^ decreased goblet cell number, goblet cell size, villi height, and intervilli space and increased villi width and smooth muscle thickness in the intestine. At 4 mpf, WT and kras+ zebrafish were treated with 2 μM of mifepristone and 40 ng/mL of LPS for 4 weeks, and samples were then collected for gross observations and histological analysis. There were four experimental groups: WT, WT/LPS, kras+, and kras+/LPS (WT, N = 20; WT/LPS, N = 20; kras+, N = 20; kras+/LPS, N = 20). (**A**) PAS staining was carried out in intestinal sections. (**B**–**G**) Quantification of goblet cell number, goblet cell size of villi, villi height, intervilli space, villi width, and smooth muscle thickness in the intestine. Differences among the variables were assessed using Student’s t-tests. Statistical significance: * *p* < 0.05, ** *p* < 0.01, *** *p* < 0.001. Scale bar: 100 μm.

To examine the effects of LPS on the intestinal morphology of WT/LPS and kras+/LPS zebrafish, we examined the number and size of goblet cells; the height, intervilli space, and width of the villi; and the thickness of smooth muscles (Figure 3A). In a 4-week induction experiment, histology analysis revealed that the number (Figure 3B) and size (Figure 3C) of goblet cells were significantly reduced in WT/LPS and kras+LPS zebrafish compared with WT, WT/LPS, and kras+ zebrafish. Intestinal villi height (Figure 3D) and intervilli space (Figure 3F) were also significantly reduced in WT/LPS zebrafish compared with WT/WT/LPS and kras+ zebrafish. Conversely, intestinal villi width (Figure 3E) and smooth muscle thickness (Figure 3G) were significantly increased in WT/LPS and kras+/LPS zebrafish. These observations indicate that *kras^V^*^12^ expression combined with LPS treatment significantly enhanced changes in intestinal morphology.

### 3.4. Increases in Cell Apoptosis, Proliferation, and Downstream Signaling of Phosphorylated AKT and ERK Induced by Sustained Expression of kras^V12^ with LPS Treatment in Transgenic Zebrafish

**Figure 4:**
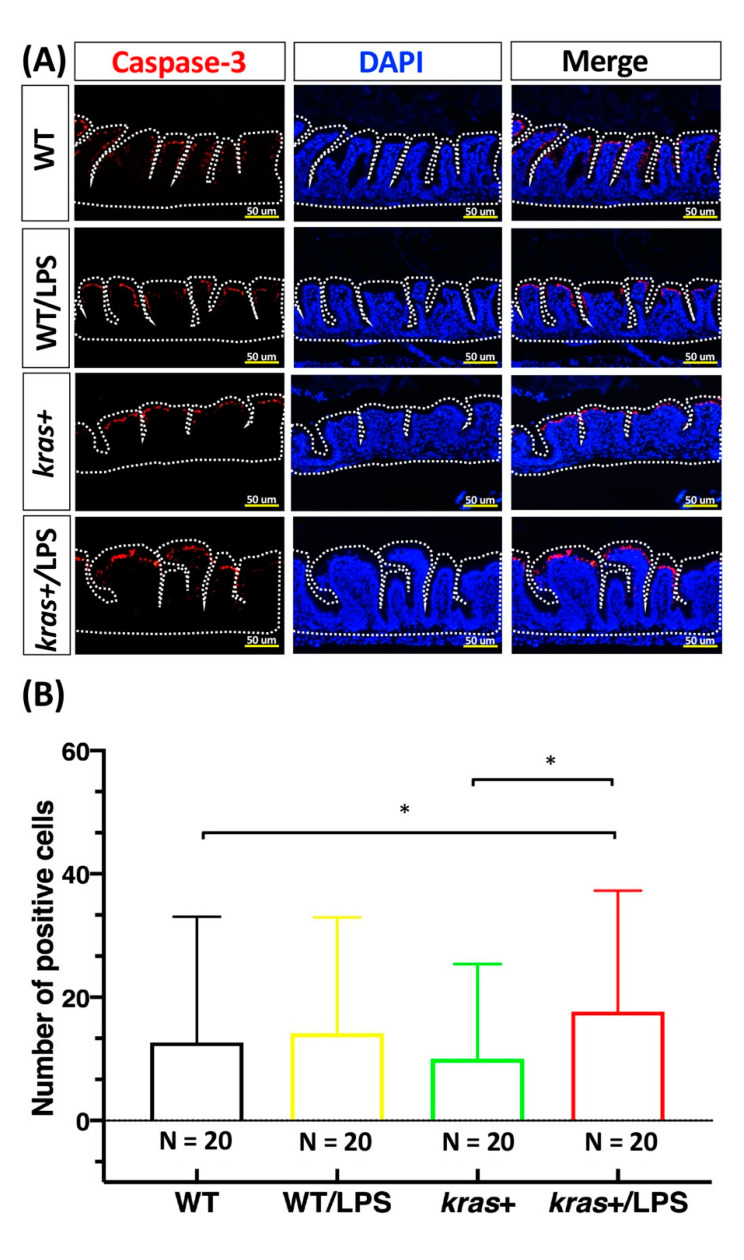

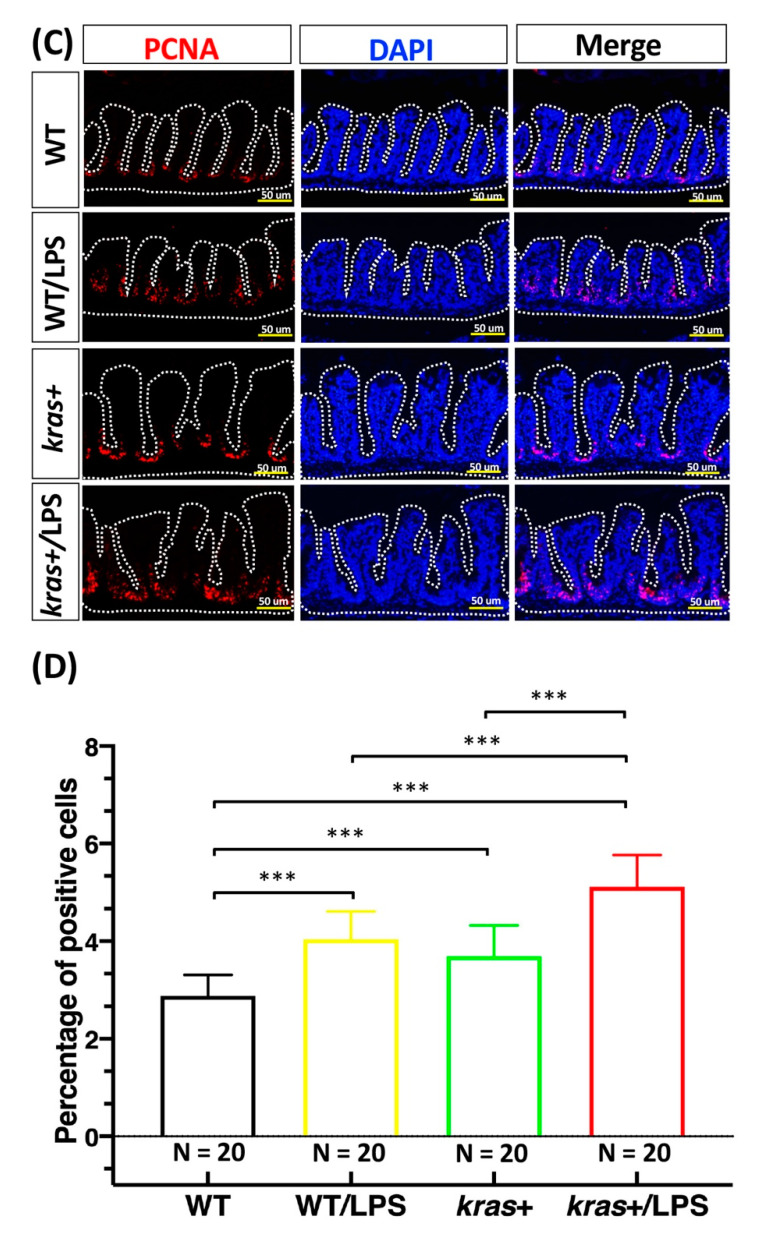
Expression of *kras^V^*^12^ with LPS treatment enhanced the increase in cell apoptosis and cell proliferation in the intestinal epithelium. (**A**,**C**) Immunofluorescence staining (red) was carried out in intestinal paraffin sections of WT (N = 20), WT/LPS (N = 20), kras+ (N = 20), and kras+/LPS (N = 20) zebrafish. (**B**,**D**) Immunofluorescence staining of caspase-3 showing (1) apoptosis and (2) PCNA as a marker for cell proliferation as well as (3) quantification of the number and percentage of positive cells. Differences among the variables were assessed using Student’s t-tests. Statistical significance: * *p* < 0.05, *** *p* < 0.001. Scale bar: 50 μm.

**Figure 5:**
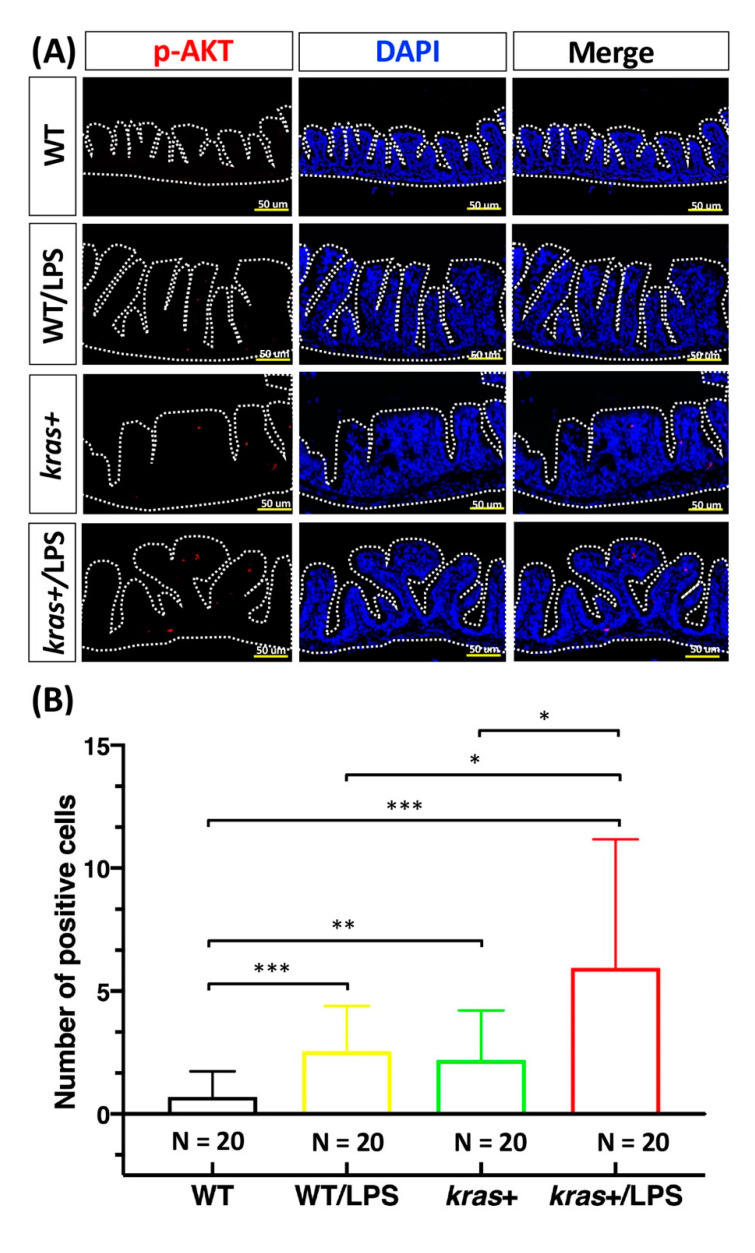

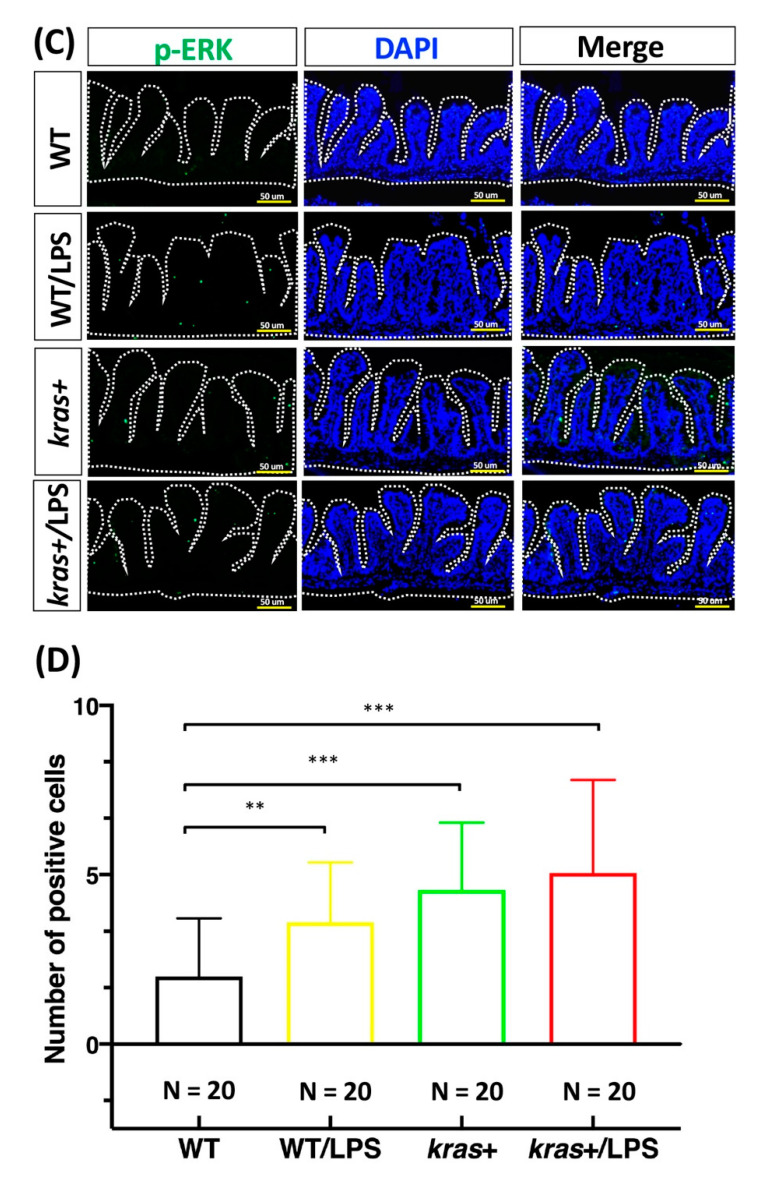
Expression of *kras^V^*^12^ with LPS treatment enhanced the increase in *p*-AKT in intestinal epithelial cells. (**A**,**C**) Immunofluorescence staining (red) was performed on intestinal paraffin sections of WT (N = 20), WT/LPS (N = 20), kras+ (N = 20), and kras+/LPS (N = 20) zebrafish. (**B**,**D**) Immunofluorescence staining of *p*-AKT and *p*-ERK as a marker of RAS signaling and quantification of the number of positive cells. Differences among the variables were assessed using Student’s t-tests. Statistical significance: * *p* < 0.05, ** *p* < 0.01, *** *p* < 0.001. Scale bar: 50 μm.

We next aimed to clarify the effects of LPS treatment on intestinal cell apoptosis and proliferation in *kras^V^*^12^-expressing zebrafish. For this, immunofluorescence staining was performed on intestine paraffin sections from WT, WT/LPS, kras+, and kras+/LPS zebrafish treated with 2 μM of mifepristone or 40 ng/mL of LPS. We found that *kras^V^*^12^-expressing zebrafish treated with LPS showed significant increases in both caspase-3 (Figure 4A,B) and PCNA-labeled cells (Figure 4C,D) compared with WT/LPS and kras+ zebrafish. In addition, *kras^V^*^12^ expression combined with LPS treatment increased the expression of a mitotic marker of phosphorylated histone (*p*-histone) in intestinal epithelial cells compared with WT/LPS and kras+ zebrafish (Figure S1). Our previous results revealed that the expression of *kras^V^*^12^ in zebrafish intestines significantly stimulated AKT and ERK activities, leading to the upregulation of the MAPK/ERK pathway (the MAPK/ERK pathway is the main downstream effector of RAS in the development of intestinal tumors) [27]. Furthermore, immunofluorescence staining for phosphorylated AKT (*p*-AKT) and ERK (*p*-ERK) revealed that compared with WT/LPS and kras+ zebrafish, *kras^V^*^12^ expression combined with LPS treatment significantly increased *p*-AKT levels (Figure 5A,B) but not *p*-ERK levels (Figure 5C,D) in intestinal epithelial cells. 

### 3.5. Cotreatment with DSS and LPS Enhanced Intestinal Inflammation in kras^V12^ Transgenic Zebrafish Larvae

**Figure 6:**
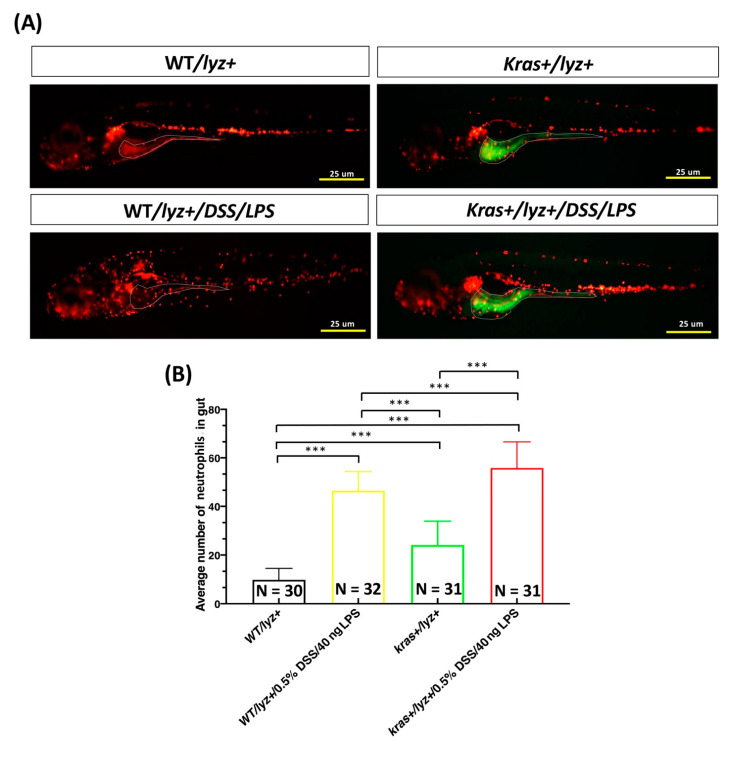

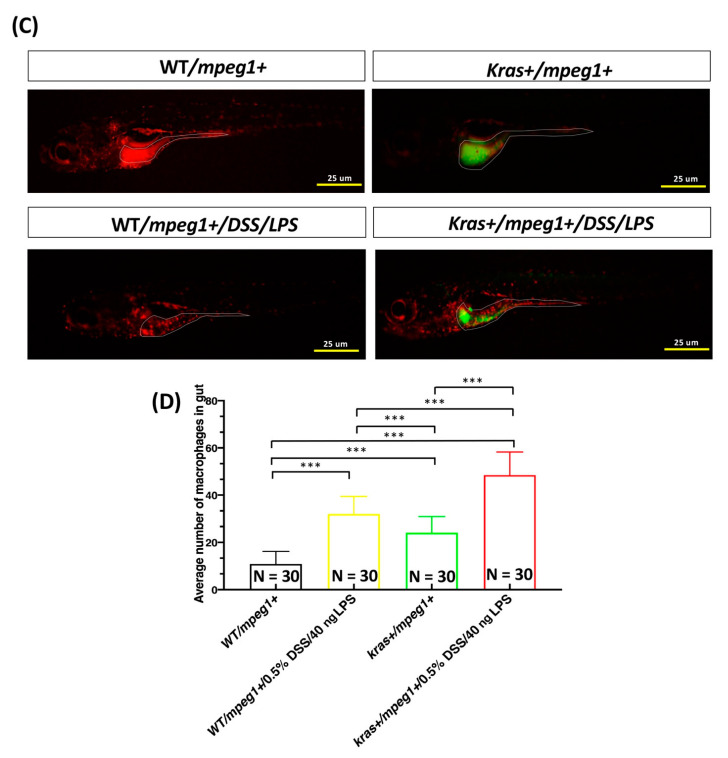
Cotreatment with DSS and LPS exacerbated the increased number of neutrophils and macrophages in the intestines of kras+/lyz+ and kras+/mepg1+ zebrafish. (**A**,**C**) Fluorescence of neutrophils (WT/lyz+, N = 30; WT/lyz+/DSS/LPS, n = 32; kras+/lyz+, n = 31; kras+/lyz+/DSS/LPS, n = 31) or macrophages (WT/mpeg1+, N = 30; WT/mepg1+/DSS/LPS, N = 30; kras+/mpeg1+, N = 30; kras+/mepg1+/DSS/LPS, N = 30) in the intestines. (**B**,**D**) Quantification of the number of positive cells as revealed by fluorescence of neutrophils or macrophages. Differences among the variables were assessed using Student’s t-tests. Statistical significance: *** *p* < 0.001. Scale bar: 25 μm.

We further tested potential synergistic effects on intestinal inflammation by cotreating WT/lyz+, kras+/lyz, WT/mpeg1+, and kras+/mpeg1+ zebrafish larvae from 4 to 6 or 7 dpf with 0.5% DSS and 40 ng/mL of LPS, in addition to treatment with 4 μM of mifepristone (to induce *kras^V^*^12^ expression) (WT/lyz+, kras+/lyz+, WT/mpeg1+, kras+/mpeg1+ without treatments served as controls). All larvae from each group were imaged. Cotreatment with LPS and DSS revealed that both numbers of neutrophils and macrophages were significantly increased in kras+/lyz+/DSS/LPS and kras+/mpeg1+/DSS/LPS larvae compared with WT/lyz+/DSS/LPS (Figure 6A,B) and WT/mpeg1+/DSS/LPS larvae (Figure 6C,D). We also observed significant increases in neutrophils and macrophages in kras+/lyz+/DSS/LPS and kras+/mpeg1+/DSS/LPS larvae compared with kras+/lyz+/LPS, kras+/lyz+/DSS, kras+/lyz+/DSS, and kras+/mpeg1+/DSS larvae (Figure S2).

### 3.6. Phenotype of Intestinal Tumors Induced by Sustained Expression of kras^V12^ with DSS and LPS Cotreatment in Transgenic Zebrafish

**Figure 7:**
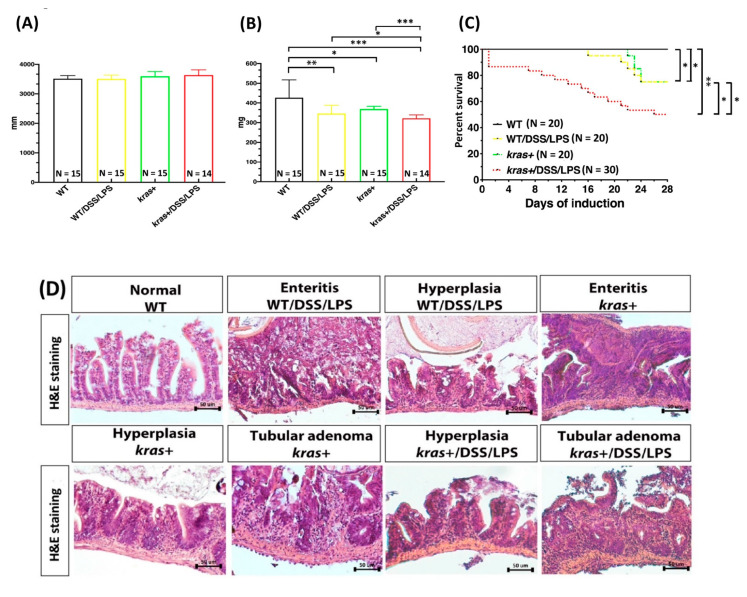

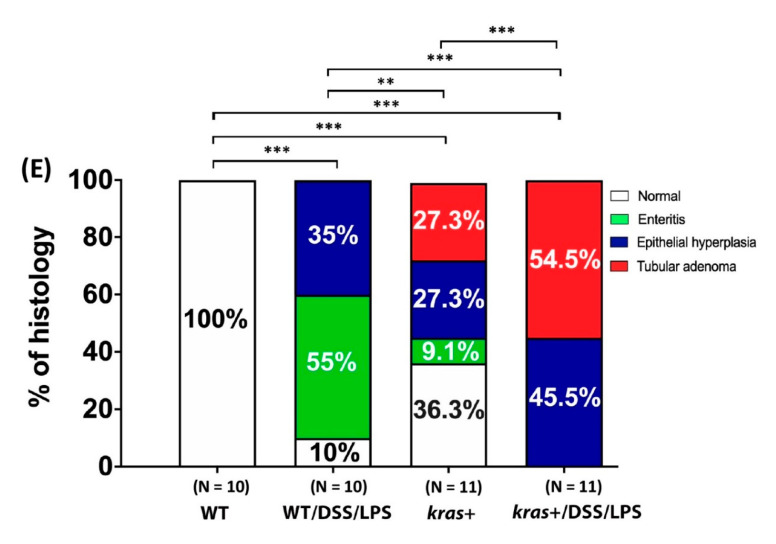
Synergistic effect of *kras^V^*^12^ expression and LPS/DSS on intestinal tumorigenesis. Four-month-postfertilization wild-type and kras+ zebrafish were cotreated with 2 μM of mifepristone, 40 ng/mL of LPS, and 0.0625% DSS for 4 weeks, and samples were then collected for gross observations and histological analyses. There were four experimental groups: WT, WT/DSS/LPS, kras+, and kras+/DSS/LPS. (**A**,**B**) Body length and body weight. (**C**) Survival curves. (**D**) Examples of normal intestines, enteritis, hyperplasia, and tubular adenoma as revealed by H&E staining of intestinal sections. (**E**) Summary of intestinal histological abnormalities observed in the four experimental groups. These data were generated as a result of a blinded histological analysis (WT, N = 10; WT/DSS/LPS, N = 10; kras+, N = 11; kras+ with DSS/LPS, N = 11). Differences among the variables were assessed using Student’s t-tests or one-way ANOVA. Statistical significance: * *p* < 0.05, ** *p* < 0.01, *** *p* < 0.001. Scale bar: 50 μm.

We further confirmed the cotreatment of DSS/LPS in *kras^V^*^12^ transgenic zebrafish at the adult stage. For this, 4 mpf heterozygous *kras^V^*^12^ zebrafish were cotreated with 2 μM of mifepristone and DSS/LPS (0.0625%; 40 ng/mL) for 4 weeks. No significant differences were found in terms of body length between kras+ zebrafish treated with DSS/LPS and control group fish (Figure 7A). However, we observed significantly reduced body weights in adult WT/DSS/LPS and kras+/DSS/LPS zebrafish compared with WT or WT/DSS/LPS zebrafish (Figure 7B).

By 4 wpi, 5 of the WT/DSS/LPS or kras+ zebrafish from each group and 13 of the kras+/DSS/LPS zebrafish had died, whereas no WT zebrafish died during the same period (Figure 7C). At 4 wpi, we evaluated the entire intestinal tract of adult zebrafish for enteritis, epithelial hyperplasia, and the presence of tumors (Figure 7D). For this, intestinal samples were collected from WT, WT/DSS/LPS, kras+, and kras+/DSS/LPS groups, and histological examinations were then performed. While all WT zebrafish showed normal intestinal histology, DSS/LPS treatment resulted in 55% and 35% of WT zebrafish respectively developing enteritis and hyperplasia. In kras+ zebrafish, mifepristone treatment led to inflammation, hyperplasia, and tubular adenoma in 9.1%, 27.3%, and 27.3% of these zebrafish, respectively. DSS/LPS treatment in kras+ zebrafish further increased the prevalence of abnormalities, whereby 45.5% and 54.5% of zebrafish respectively exhibited hyperplasia and tubular adenoma. These observations suggest that *kras^V^*^12^ expression combined with DSS/LPS treatment significantly increased the prevalence of intestinal tumors in adult zebrafish compared with WT zebrafish treated with DSS/LPS (Figure 7E). We also confirmed the WT or kras+ cotreatment of DSS/LPS at the adult stage of zebrafish compared with WT/LPS or kras+/LPS. Results from WT/DSS/LPS and kras+/DSS/LPS transgenic zebrafish were not significantly different from those of WT/LPS and kras+/LPS control groups (Figure S3). 

## 4. Discussion

The development of CRC is a multistep process that involves the progression of normal epithelium to an adenoma and then to an adenocarcinoma, where the adenocarcinoma may eventually metastasize to different organs [35]. The development a genetic model of colorectal tumorigenesis was introduced in 1990. In this model, APC, KRAS, TP53, and DCC were proposed as genes that promote CRC progression [36]. Since the introduction of this model, many potential molecular mechanisms of CRC have been investigated. There is a consensus that CRC development is related to LPS-induced systemic inflammation, and these events alter the signal transduction of TLR4, NF-κB, and transforming growth factor beta 1 (TGF-β1) pathways [17,37]. In mice, LPS has been found to contribute to tumor progression and hepatic metastasis of colon cancer cells [17,38,39]. Furthermore, the DSS-induced colitis model is widely used because it has many similarities with human ulcerative colitis [40]. Furthermore, a huge advantage of transgenic zebrafish can be an exceptional platform for mimicking human intestinal disorder and establishing vertebrate models for drug screening. For intestinal disease research, high tumor incidence and convenient chemical treatment make the inducible transgenic zebrafish a reasonable platform for intestinal inflammation and tumor progression [23,27]. However, no previously published studies have reported that *kras^V^*^12^ expression combined with LPS treatment can induce intestinal tumors in zebrafish. This is also the first study to report that LPS and/or DSS treatment promotes intestinal tumor progression in *kras^V^*^12^-expressing transgenic zebrafish.

Tumor-associated macrophages and tumor-associated neutrophils are among the most abundant immune cells in the tumor microenvironment. In CRC, they play pivotal tumor-supporting roles [41,42]. In this study, a zebrafish model was used to study the effects of LPS and/or DSS treatment on intestinal inflammation in *kras^V^*^12^ transgenic zebrafish larvae. Specifically, we were interested in (1) the effects that *kras^V^*^12^ expression has on neutrophils and macrophages when combined with LPS and/or DSS treatment and (2) how these effects stimulate the immune system (Figure 1). We previously found that treating *kras^V^*^12^ zebrafish larvae with LPS led to significant increases in neutrophil count and neutrophil density in the liver. These increases in neutrophils further led to an enlargement in liver size [43]. In adult zebrafish, the intestine-specific expression of *kras^V^*^12^ with LPS treatment also led to an increase in hyperplasia and tubular adenoma (Figure 2D,E). In addition, intestinal morphology (Figure 3A) revealed that goblet cell number, goblet cell size, villi height, intervilli space, villi width, and smooth muscle thickness (Figure 3B–G) were also significantly altered in these *kras^V^*^12^ zebrafish. Goblet cells in intestinal epithelium produce and secrete mucins (predominantly MUC2), which enter the intestinal lumen to form a mucus layer [44]. The mucus and mucins of goblet cells and intestinal epithelial cells compose the first line of defense for the gastrointestinal tract and interact with the immune system [45]. In clinical CRC samples from mice, SCF/c-KIT signaling has been shown to promote mucus secretion in colonic goblet cells as well as the development of mucinous colorectal adenocarcinoma [46]. CRC tumors and cell lines are characterized by an increased expression of goblet cell marker genes, and there is an association between the proportion of goblet cells in a tumor and the probability that the tumor is assigned as consensus molecular subtype 3 (CMS3) (CMS3 is a tumor subtype that is mutually exclusive from mucinous adenocarcinoma pathologies [47]). Changes in mucin gene expression and mucin glycan structure can occur in intestinal cancers, which leads to cancer progression [48]. Our results indicated that *kras^V^*^12^ and/or LPS can critically interact with the immune system and may be involved in the development of intestinal carcinogenesis.

We analyzed intestinal tumor formation using histological and immunochemical methods. Immunochemical data showed an increase in active caspase-3 (Figure 4A,B), PCNA expression (Figure 4C,D), and downstream signaling of phosphorylated AKT (Figure 5A,B) in kras+/LPS zebrafish compared with kras+ zebrafish, which suggests that LPS is associated with intestinal tumor formation. LPS has been reported to cause rapid apoptosis and death in intestinal epithelial cells as well as loss of epithelial integrity, which is contingent on apoptosis functioning normally [49,50,51]. LPS treatment has also been found to increase (1) AKT phosphorylation at residue Ser473 and (2) increase liver metastasis of HT-29 cells, both in vitro and in vivo [17]. Our data indicate that *kras^V^*^12^ and LPS can be linked through the AKT pathway. The AKT pathway has roles in apoptosis [52], cell proliferation, cell migration [53], angiogenesis [54], and metabolism [55]. Thus, this *kras*/LPS/AKT link may be the mechanism that underlies the development of intestinal carcinogenesis in *kras^V^*^12^ transgenic zebrafish.

Recently, inflammation has been found to increase the risk of CRC [56,57,58]. DSS has been shown to induce inflammation of the colonic mucosa and has a tumor-promoting effect in mouse and zebrafish models [27,59]. Moreover, a DSS-induced inflammatory bowel disease (IBD)-like enterocolitis model has also been established in zebrafish [60]. Inflammatory stimuli induced by DSS treatment following initiation with AOM carcinogens are effective at rapidly inducing inflammation and intestinal tumors [61]. In mice, high-fat diets can also promote colon tumors associated with AOM/DSS-induced colitis [62]. In addition, DSS treatment has been found to initiate the development of small intestinal polyps or tumors at 2% and 4%, respectively, in mouse models [63,64]. Therefore, we further investigated whether cotreatment with DSS/LPS can exacerbate intestinal inflammation associated with neutrophils or macrophages in *kras^V^*^12^ transgenic zebrafish larvae. In *kras^V^*^12^ zebrafish larvae, DSS/LPS treatment led to significant increases in both of neutrophil and macrophage numbers in the intestines (Figure 6). In addition, LPS/DSS cotreatment significantly enhanced the increase in neutrophils and macrophages in the intestines in kras+/lyz+/LPS and kras+/lyz+/DSS as well as in kras+/mpeg1+/LPS and kras+/mpeg1+/DSS zebrafish during the larval stage (Figure S2). For adult zebrafish, *kras^V^*^12^ expression combined with DSS/LPS cotreatment led to an increase in the prevalence of hyperplasia and tubular adenoma compared with WT/DSS/LPS adult zebrafish (Figure 7D,E). Therefore, the current research also strongly supports a relationship between chronic inflammation and intestinal tumorigenesis. However, we did not observe significant differences in intestinal tumorigenesis between WT/LPS and WT/LPS/DSS or between kras+/LPS and kras+/DSS/LPS adult-stage zebrafish (Figure S3). These results indicate that extending the treatment time may be necessary to strengthen the state of chronic inflammation [65,66].

## 5. Conclusions

In conclusion, our results (based on zebrafish treated with LPS alone or cotreated with LPS and DSS) provide evidence that LPS and/or DSS exacerbates the development and progression of intestinal tumors in *kras^V^*^12^ transgenic zebrafish. These findings are an extension of our previous data [27], which showed that DSS increased *kras^V^*^12^-induced intestinal tumors in zebrafish. The current study demonstrated that *kras^V^*^12^ expression combined with LPS and/or DSS treatment also significantly exacerbated the development and progression of intestinal tumors, and this is achieved through the modulation of the PI3K–AKT signaling pathway. Therefore, further research is necessary to explore the effects of other inflammatory agents on CRC progression in humans.

## Data Availability

Data are contained within the article.

## Supplementary Materials

The following are available online at https://www.mdpi.com/article/10.3390/biomedicines9080974/s1: Figure S1: Expression of *kras^V^*^12^ with LPS treatment enhanced the increase in p-Histone in intestinal epithelial cells, Figure S2: LPS/DSS co-treatment significantly enhanced the increase number in neutrophils and macrophages in the intestine during the larval stage in kras+/lyz+/LPS and kras+/lyz+/DSS as well as in kras+/mpeg1+/LPS and kras+/mpeg1+/DSS zebrafish, Figure S3: No significant synergistic effects on intestinal tumorigenesis between WT/LPS and WT/LPS/DSS or between kras+/LPS and kras+ with DSS/LPS adult stage zebrafish, Table S1: Information of antibodies used in this study.
