# Supplementary material for: Lipopolysaccharides Enhance Epithelial Hyperplasia and Tubular Adenoma in Intestine-Specific Expression of krasV12 in Transgenic Zebrafish
Source: Biomedicines. 2021 Aug 7;9(8):974. doi: 10.3390/biomedicines9080974 (PMC8393945; doi:10.3390/biomedicines9080974)
Supplement: Supplementary file 1 [file biomedicines-09-00974-s001.zip › biomedicines-1302596-supplementary.pdf]

biomedicines

Supplementary materials

Lipopolysaccharides Enhance Epithelial Hyperplasia and Tubular Adenoma in Intestine-Specific Expression of $kras^{V12}$ in Transgenic Zebrafish

Jeng-Wei Lu [1,2,*], Yuxi Sun [1,3], Pei-Shi Angelina Fong [1], Liang-In Lin [2,4], Dong Liu [3] and Zhiyuan Gong [1,*]

Table S1. Information of antibodies used in this study

Name	Catalog number	Dilution	Company
Rabbit anti-pcna	GTX124496	200	GeneTex, CA, USA
Rabbit anti-Cleaved Caspase-3(D175)	9661S	200	Cell Signaling Technology, Danvers, MA, USA
Rabbit p-AKT (S473)	4060S	200	Cell Signaling Technology, Danvers, MA, USA
Anti-MAP Kinase	M9692	100	Sigma, St. Louis, MO, USA
Rabbit anti-Histone H3	ab5176	200	Abcam, Cambridge, UK
Alexa Fluor 546 Donkey anti-rabbit	A10040	200	Invitrogen, Carlsbad, CA, USA
Alexa Fluor 488 Donkey anti-mouse	A21202	200	Invitrogen, Carlsbad, CA, USA

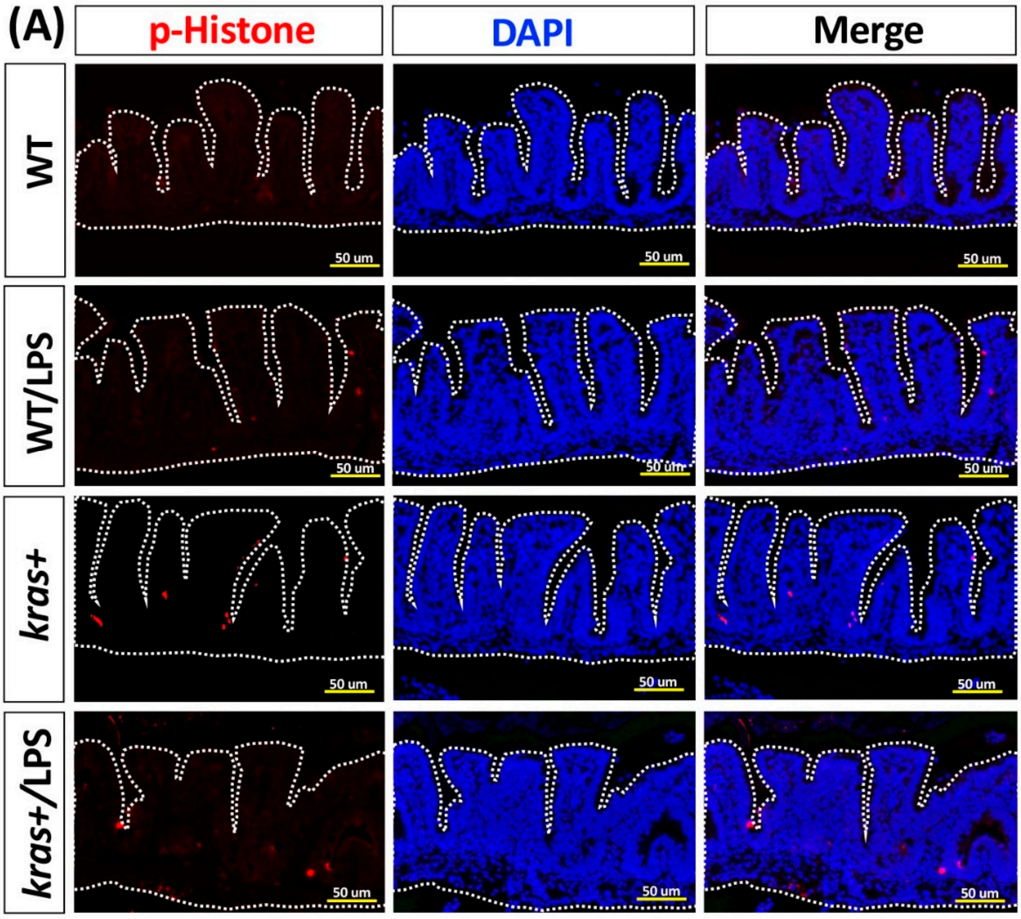
(A)
p-Histone
DAPI
Merge
WT
WT/LPS
kras+
kras+/LPS
50 um

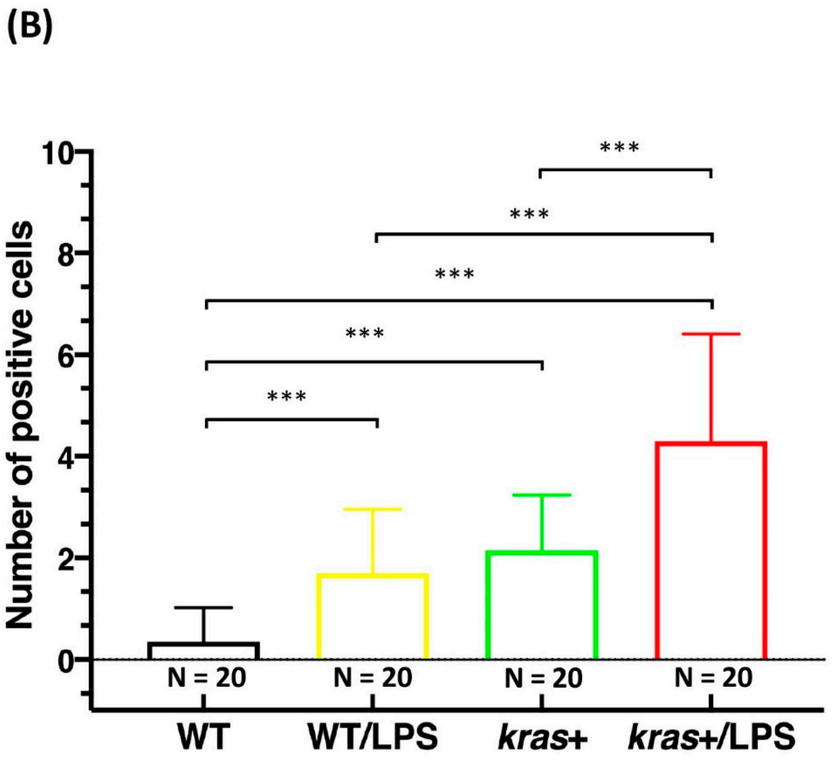

Figure S1. Expression of *kras*^{V12} with LPS treatment enhanced the increase in p-Histone in intestinal epithelial cells. **(A)** Immunofluorescence staining (red) was carried out in intestinal paraffin sections of WT (n= 20), WT/LPS (n= 20), kras+ (n= 20), and kras+/LPS (n= 20) zebrafish. **(B)** Immunofluorescence staining of p-Histone as a marker of mitosis, and quantification of the number of positive cells. Differences among the variables were assessed using Student's t-tests. Statistical significance: *P<0.05, **P<0.01, ***P<0.001. Scale bar: 50 μm.

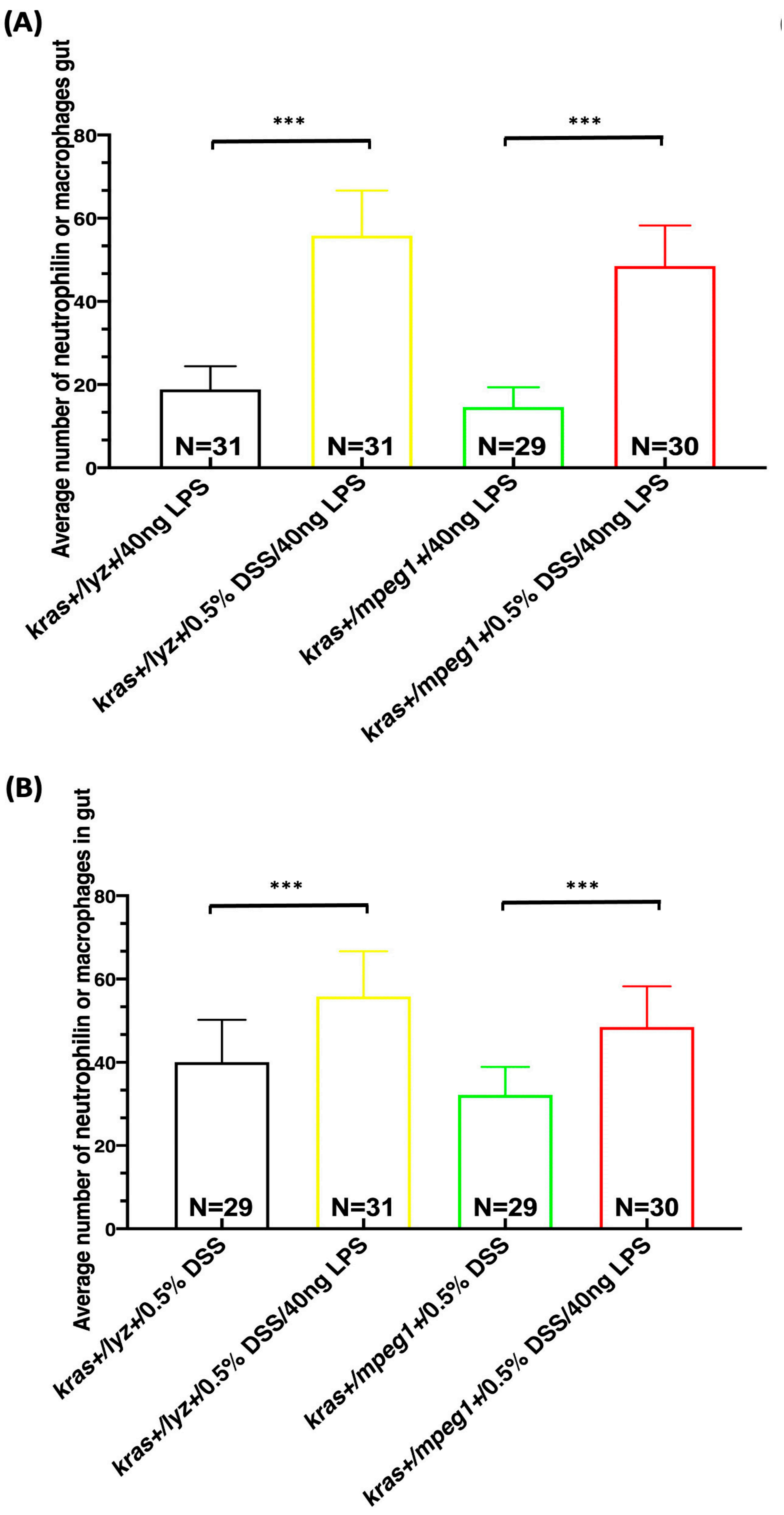
(A)
Average number of neutrophilin or macrophages gut
0
20
40
60
80
***
***
N=31
N=31
N=29
N=30
kras+/lyz+/40ng LPS
kras+/lyz+/0.5% DSS/40ng LPS
kras+/mpeg1+/40ng LPS
kras+/mpeg1+/0.5% DSS/40ng LPS
(B)
Average number of neutrophilin or macrophages in gut
0
20
40
60
80
***
***
N=29
N=31
N=29
N=30
kras+/lyz+/0.5% DSS
kras+/lyz+/0.5% DSS/40ng LPS
kras+/mpeg1+/0.5% DSS
kras+/mpeg1+/0.5% DSS/40ng LPS

Figure S2. LPS/DSS co-treatment significantly enhanced the increase number in neutrophils and macrophages in the intestine during the larval stage in kras+/lyz+/LPS and kras+/lyz+/DSS as well as in kras+/mpeg1+/LPS and kras+/mpeg1+/DSS zebrafish. **(A and B)** Quantification of the number of positive cells as revealed by fluorescence of neutrophils or macrophages in the intestine (kras+/lyz+/LPS, n= 31; kras+/lyz+/DSS, n= 29; kras+/lyz+/LPS/DSS, n= 31; kras+/mpeg1+/LPS, n= 29; kras+/mpeg1+/DSS, n= 29; kras+/mpeg1+/LPS/DSS, n= 30). Differences among the variables were assessed using Student's t-tests. Statistical significance: *P<0.05, **P<0.01, ***P<0.001.

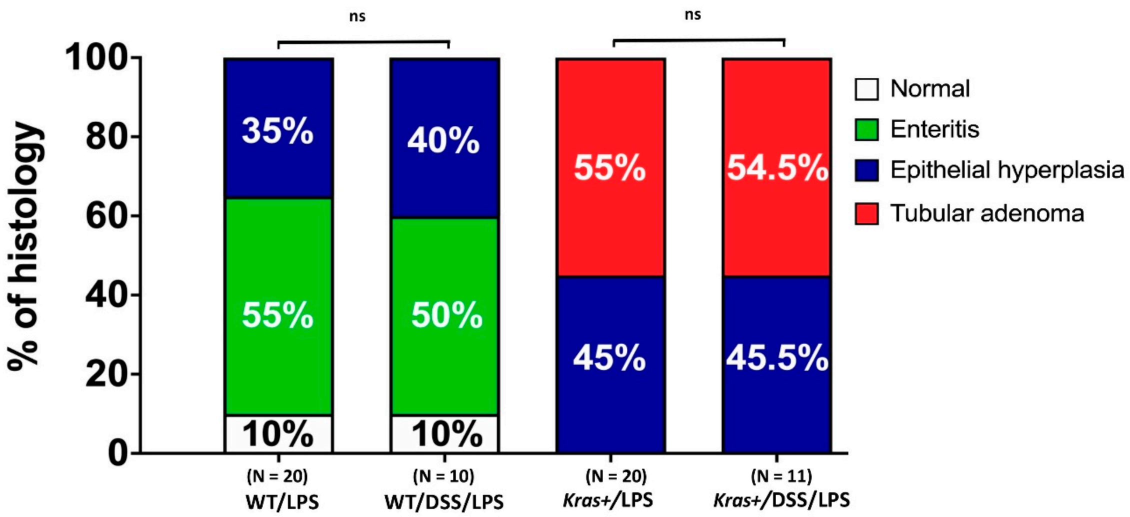

Figure S3. No significant synergistic effects on intestinal tumorigenesis between WT/LPS and WT/LPS/DSS or between kras+/LPS and kras+ with DSS/LPS adult stage zebrafish. Summary of intestinal histological abnormalities observed in the four experimental groups. The data were generated from results of a blinded histological analysis (WT/LPS, n= 20; WT/DSS/LPS, n= 10; kras+/LPS, n= 11; kras+ with DSS/LPS, n= 11). Differences among the variables were assessed using one-way ANOVA. Statistical significance: *P<0.05, **P<0.01, ***P<0.001.
